# Anti-Cancer Effects of Glaucarubinone in the Hepatocellular Carcinoma Cell Line Huh7 via Regulation of the Epithelial-To-Mesenchymal Transition-Associated Transcription Factor Twist1

**DOI:** 10.3390/ijms22041700

**Published:** 2021-02-08

**Authors:** Jihye Seo, Jain Ha, Eunjeong Kang, Haelim Yoon, Sewoong Lee, Shi Yong Ryu, Kwonseop Kim, Sayeon Cho

**Affiliations:** 1 Laboratory of Molecular and Pharmacological Cell Biology, College of Pharmacy, Chung-Ang University, Seoul 06974, Korea; seojh0228@gmail.com (J.S.); joehalee@gmail.com (J.H.); ejaykang@gmail.com (E.K.); limtiny@naver.com (H.Y.); dltpdnd2000@naver.com (S.L.); 2 Korea Research Institute of Chemical Technology (KRICT), Daejeon 34114, Korea; syryu@krict.re.kr; 3 College of Pharmacy and Research Institute for Drug Development, Chonnam National University, Gwangju 61186, Korea; koskim@jnu.ac.kr

**Keywords:** glaucarubinone, Twist1, epithelial-to-mesenchymal transition, anti-cancer

## Abstract

Hepatocellular carcinoma (HCC), the most common type of liver cancer, is a leading cause of cancer-related deaths. As HCC has a high mortality rate and its incidence is increasing worldwide, understanding and treating HCC are crucial for resolving major public health concerns. In the present study, wound healing screening assays were performed using natural product libraries to identify natural chemicals that can inhibit cancer cell migration. Glaucarubinone (GCB) showed a high potential for inhibiting cell migration. The anti-cancer effects of GCB were evaluated using the HCC cell line, Huh7. GCB showed anti-cancer effects, as verified by wound healing, cell migration, invasion, colony formation, and three-dimensional spheroid invasion assays. In addition, cells treated with GCB showed suppressed matrix metalloproteinase activities. Immunoblotting analyses of intracellular signaling pathways revealed that GCB regulated the levels of Twist1, a crucial transcription factor associated with epithelial-to-mesenchymal transition, and mitogen-activated protein kinase. The invasive ability of cancer cells was found to be decreased by the regulation of Twist1 protein levels. Furthermore, GCB downregulated phosphorylation of extracellular signal-regulated kinase. These results indicate that GCB exhibits anti-metastatic properties in Huh7 cells, suggesting that it could be used to treat HCC.

## 1. Introduction

Liver cancer is the fifth most common type of cancer in men and the eighth most common type of cancer in women worldwide [1]. Most cancer-related deaths are not caused by primary cancers but by metastasis, the process by which cancer cells spread to peripheral organs [2,3]. Invasive and motile cancer cells can enter the circulatory system even before they form tumors; hence, the development of malignant tumors is sometimes known as the beginning of metastasis. Most cancer cells that enter the circulatory system perish; however, even a small percentage of cells that survive can infiltrate distant organs [4]. Epithelial-to-mesenchymal transition (EMT) is the most critical process in metastasis; in EMT, cancer cells lose their polarity and separate from each other, adopt the characteristics of the mesenchymal phenotype, become motile, and invade distant sites. E-cadherin, the most important EMT marker, is a cell–cell adhesion molecule; the loss of E-cadherin expression increases cell mobility and promotes tumor cell invasion [5,6,7]. Major EMT-associated transcription factors, such as Snail1/2, Zeb1/2, and Twist1, are the transcription inhibitors of E-cadherin [8].

Twist and Snail families not only reduce E-cadherin expression and increase matrix metalloproteinase (MMP) expression but also play a role in the invasion of a wide variety of cancer cell types [9]. The decomposition of the extracellular matrix and destruction of the basement membrane by MMPs enable cancer cell invasion and ultimately lead to a critical step involved in metastasis [10]. The expression and stability of these EMT-associated transcription factors are regulated by diverse intracellular signaling pathways. In particular, the mitogen-activated protein kinase (MAPK) pathways, including the extracellular signal-regulated kinase (ERK), c-Jun N-terminal kinase (JNK), and p38 pathways, play a vital role in the growth and development of many human cancer cells [11]. Specifically, the MAPK/ERK (Ras–Raf–MEK–ERK) pathway regulates the stability of Twist1 by phosphorylation at Ser68 [12]. When Twist1 is phosphorylated at Ser68 by MAPK, its stability increases, resulting in the elevation of Twist1 protein levels [12]. Studies focus on targeting cancer by compounds that regulate the signaling pathways in various types of cancer. In particular, efforts are being made to control the metastatic properties of cancer cells by regulating their transcription factors.

Glaucarubinone (GCB) was first reported as one of the quassinoids originally isolated from the stem bark of *Ailanthus triphysa* [13]. It has been found to inhibit the activity of activator protein 1 (AP-1) stimulated by 12-O-tetradecanoylphorbol 13-acetate [13]. Moreover, GCB exhibits anti-cancer effects by regulating p21-activated kinases (PAKs) [14,15]. Although GCB has been shown to have anti-cancer effects, the mechanisms underlying its anti-migratory effects remain unclear. Therefore, in the present study, we further evaluated the potential of GCB as an anti-cancer agent by assessing the mechanisms underlying its anti-migratory effects in liver cancer cells.

## 2. Results

### 2.1. GCB Showed Low Cytotoxicity

**Figure 1:**
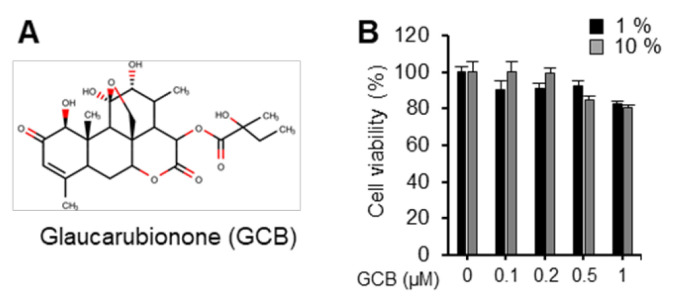
GCB was one of the top candidates without significant cytotoxicity. (**A**) Chemical structure of GCB. (**B**) Huh7 cells were treated with GCB at the indicated concentration in the cell culture medium containing 1% or 10% FBS for 24 h. The cell viability was measured using the EZ-CYTOX solution. The relative cell viability is shown using bar graphs in comparison with the untreated control (100%). Data are representative of three experiments and are expressed as the mean ± SEM.

Screening through wound healing screening assays were performed using a total of 1100 natural chemical compounds obtained from the Korea Chemical Bank (KCB) at a chemical concentration of 1 μM. After three repeats of screening, information on the primary positive candidates was obtained from the KCB. Several chemicals previously known to regulate metastasis signalings were identified, including vinblastine, bufalin, tomatine, homoharringtonine, and mogroside IV [16,17,18,19,20], thus, underscoring the validity of our screening. Cytotoxicity tests were performed using novel candidate chemicals to exclude chemicals that could induce strong cell death. GCB (Figure 1A) was selected for further analyses as it consistently showed strong inhibition of Huh7 cell migration and relatively low cytotoxicity. Since media containing 1% and 10% FBS were used in subsequent experiments, the cytotoxicity of Huh7 by GCB was monitored under these conditions. On assessing the cytotoxicity of GCB in Huh7 cells, we found that GCB did not show significant cytotoxicity up to 1 μM (Figure 1B). In other liver cancer cell lines, SK-Hep1 and Hep3B, the cytotoxicity of GCB was measured and was not toxic at concentrations used in this study (Figure S1A). As sub-micromolar concentrations of GCB were not toxic to cells, the mechanisms underlying its anti-migratory effects were further investigated.

### 2.2. Malignant Traits of Huh7 Cells Were Suppressed by GCB

**Figure 2:**
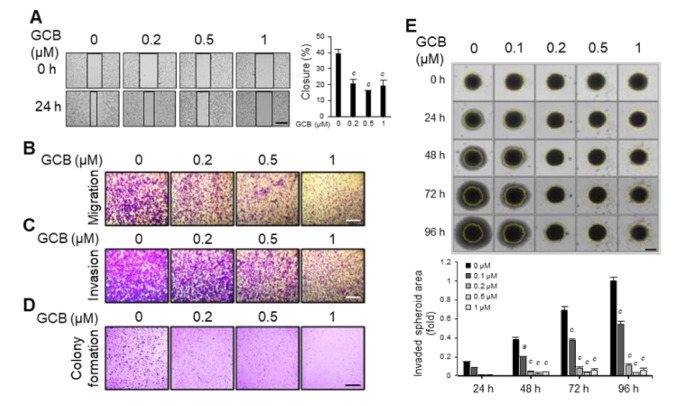
GCB could inhibit cancer cell migration and invasion. (**A**) Huh7 cells were incubated with GCB (0, 0.2, 0.5, and 1 μM). Microscopic images were taken at 24 h (scale bars, 0.5 mm). The wound closure values were quantified by measuring the percentage of wound closure in comparison with the 0 h point for each sample; the relative wound closure is shown as a bar graph. Data were analyzed by one-way ANOVA; ^c^ *p* < 0.001 relative to the untreated control. (**B**) Huh7 cells were seeded in the upper chambers of transwell plates and treated with GCB (0, 0.2, 0.5, and 1 μM) for 18 h. Images of the cells that had migrated to the other side of the membrane were taken (scale bars, 0.5 mm). (**C**) Huh7 cells were seeded with the indicated concentration of GCB in the upper chambers coated with Matrigel. After incubation for 18 h, images of the invaded cells were taken (scale bars, 0.5 mm). (**D**) Huh7 cells were incubated with different concentrations of GCB (0, 0.2, 0.5, and 1 μM). The colonies were stained with 0.5% crystal violet, and the images were then taken (scale bars, 1 cm). (**E**) Three-dimensional spheroidal Huh7 cells were incubated with GCB (0, 0.1, 0.2, 0.5, and 1 μM) for 96 h. Microscopic images were captured using a JuLI Stage Real-Time Cell History Recorder at the indicated time points (scale bars, 0.4 mm). The yellow line indicates the outline of the spheroid at 0 h. The invaded spheroid area (fold) indicates the invaded area of a spheroid at each time in comparison with the size of the spheroid at 0 h. The invaded area was measured using ImageJ. Data were analyzed by one-way ANOVA; ^a^ *p* < 0.05 and ^c^ *p* < 0.001 relative to the GCB-untreated control.

Given that cancer cells have higher motility and invasiveness [21], the effects of GCB on Huh7, SK-Hep1, and Hep3B cell motility were evaluated by a wound healing assay. Compared with the untreated cells, wound closures were suppressed when the cells were treated with increasing concentrations of GCB (Figure 2A and Figure S1B). Cell migration assays were performed using hanging transwell inserts over a short period to exclude the interference of cell growth with cell migration. The cells treated with GCB showed much lesser migration than those treated with dimethyl sulfoxide (DMSO) only (Figure 2B). As aggressive cancer cells not only have higher motility but also have invasive physiology [22], Matrigel invasion assays were performed on hanging transwell inserts. GCB treatment suppressed cell invasion through Matrigel (Figure 2C). One of the characteristics of highly metastatic cancer cells is anchorage-independent growth [23]. Colony formation assays were performed to confirm whether GCB suppresses anchorage-independent growth properties of cancer cells (Figure 2D). Anchorage-independent colony formation of Huh7 cells was suppressed by GCB in a dose-dependent manner. A three-dimensional spheroid invasion assay was performed to confirm the effect of GCB under conditions similar to those of early cancer metastasis in vivo (Figure 2E). Comparison of the control and GCB groups up to 96 h revealed that GCB suppressed the invasiveness of spheroids in a time- and dose-dependent manner. Moreover, when the spheroids were incubated with over 0.2 μM of GCB, their invasion was almost completely repressed, indicating that GCB has the potential to inhibit the invasion of cancer cells in vivo. These data suggest that GCB exhibits anti-migratory and anti-invasion effects in Huh7 cells.

### 2.3. GCB Altered MMP Activities

**Figure 3:**
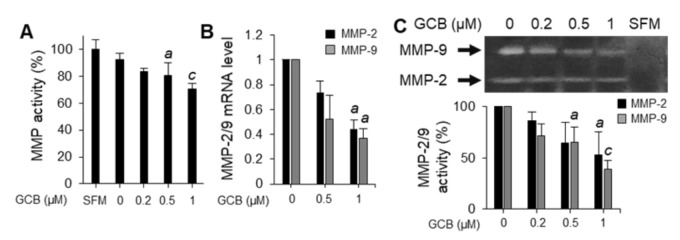
GCB inhibited MMP activity and transcription. (**A**) Gelatinase/collagenase assays were performed using secreted medium from GCB-treated Huh7 cells. Serum-free medium (SFM) was used as the negative control. The relative MMP activity was evaluated with the measured fluorescence value and is shown as a bar graph in comparison with the negative control (100%). Data were analyzed by one-way ANOVA; ^a^ *p* < 0.05 and ^c^ *p* < 0.001 relative to the negative control. (**B**) Huh7 cells were treated with GCB (0, 0.5, and 1 μM) for 24 h, and the relative mRNA expression levels of MMP-2 and -9 were measured by qRT-PCR. The *GAPDH* gene was used as the reference gene for normalization. Values are expressed as the mean ± SEM (n = 3). Data were analyzed by one-way ANOVA; ^a^ *p* < 0.05 relative to the GCB-untreated control. (**C**) Huh7 cells were incubated with GCB (0, 0.2, 0.5, and 1 μM) for 24 h in SFM. Samples were analyzed by zymography; SFM was used as the negative control. The data represent three experiments and are expressed as the mean ± SEM (n = 3). One-way ANOVA was used for analyzing the data; ^a^ *p* < 0.05 and ^c^ *p* < 0.001 relative to the GCB-untreated control.

Collagenase activity assays were performed to evaluate the activities of MMPs (MMP-1, -2 and -9) that were secreted from GCB-treated Huh7 cells and could degrade the extracellular matrix around the cancer cells. Collagen degradation by MMPs decreased with an increase in the GCB concentration (Figure 3A). MMP-2 and -9 are representative and the most well-studied MMPs in HCC [24]. In particular, MMP-2 and -9 secretion is increased in several types of human cancer, and their increased expression is associated with poor prognosis [25]. Assessment of the effects of GCB on MMPs by real-time quantitative reverse transcription polymerase chain reaction (qRT-PCR) revealed that GCB decreased the expression levels of *MMP2* and *MMP9* genes in a dose-dependent manner (Figure 3B). Moreover, gelatin zymography assays were performed to assess the effects of GCB on the activities of MMPs. When Huh7 cells were treated with GCB, the activities of MMP-2 and -9 were suppressed (Figure 3C). These results indicate that GCB suppresses the mRNA expression of MMP-2 and -9.

### 2.4. Twist1 and ERK Activities Were Inhibited by GCB

**Figure 4:**
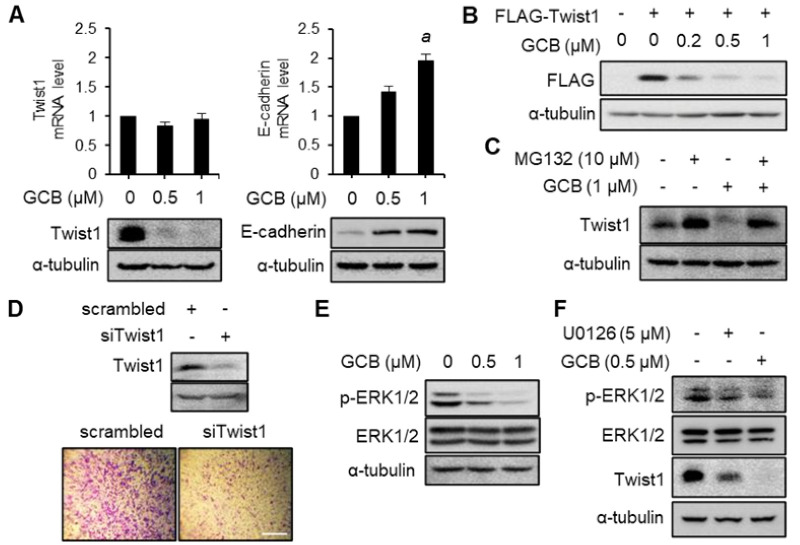
GCB treatment regulated ERK signaling and Twist1 levels. (**A**) After 24 h of incubation with GCB (0, 0.5 and 1 μM), total cell lysates were analyzed by immunoblotting (bottom panels) and qRT-PCR (top panels) for assessing the expression levels of Twist1 and E-cadherin. The mRNA expression levels of Twist1 and E-cadherin were measured and normalized using the *GAPDH* gene. The expression levels of Twist1, E-cadherin, and α-tubulin proteins were assessed using specific antibodies. Values are expressed as the mean ± SEM (n = 3). Data were analyzed by one-way ANOVA; ^a^ *p* < 0.05 relative to the GCB-untreated control. (**B**) FLAG-tagged Twist1 was overexpressed in Hep3B cells; the cells were then treated with GCB for 24 h. The expression level of Twist1 was assessed using specific antibodies. (**C**) Huh7 cells were pretreated with MG132 for 6 h and then treated with both GCB and MG132 for additional 3 h. The expression levels of Twist1 and α-tubulin proteins were assessed using specific antibodies. (**D**) Hep3B cells were transfected with siTwist1 or scrambled (control) siRNA, and the migratory activity was evaluated using transwell migration assays. Images were taken 24 h after the cells were seeded in the upper chamber (scale bars, 0.5 mm). (**E**) Huh7 cells were treated with GCB (0, 0.5, and 1 μM) for 24 h. Total cell lysates were separated by SDS–PAGE. The levels of p-ERK (Thr202/Tyr204), ERK, and α-tubulin proteins were assessed using specific antibodies. (**F**) Proteins expressed in cells treated with U0126 and GCB for 24 h were separated by SDS–PAGE, and the levels of p-ERK, ERK, Twist1, and α-tubulin proteins were detected using specific antibodies.

To further evaluate the anti-metastatic effects of GCB, the expression level of the EMT-associated transcription factor Twist1 was evaluated. When the cells were treated with GCB, Twist1 protein levels dramatically decreased (Figure 4A, left bottom panel). GCB inhibited endogenous Twist1 protein expression in a dose-dependent manner. The Twist1 protein almost disappeared at 1 μM of GCB. The mRNA levels of Twist1 were assessed to determine whether GCB suppressed Twist1 protein level via the transcriptional expression of Twist1 (Figure 4A, left top panel). The transcriptional expression of Twist1 was not inhibited by GCB, suggesting that GCB regulates Twist1 at the protein level. As Twist1 suppresses E-cadherin expression, the mRNA and protein levels of E-cadherin were assessed (Figure 4A, right panels). As expected, the expression of E-cadherin was inhibited at both transcriptional and translational levels. Twist1 ectopically overexpressed in another hepatocellular carcinoma cell line, Hep3B, was also inhibited by GCB treatment (Figure 4B). Taken together, these data suggest that the decrease in Twist1 protein levels by GCB is associated with the protein stability. Twist1 protein levels were evaluated by MG132 treatment in order to assess the effects of GCB on Twist1 protein stability (Figure 4C). The decrease in Twist1 protein levels caused by GCB was suppressed in the experimental group that was treated with MG132 to prevent proteasome-mediated degradation. As shown in Figure 4A (left panels), GCB treatment reduced only the protein level and not the mRNA level of Twist1, suggesting that decreased Twist1 protein levels result from the post-translational modification of Twist1. To identify the effects of Twist1 on cell migration, Twist1 short interfering RNA (siRNA) was transfected into Hep3B cells and transwell migration assays were performed (Figure 4D). Twist1 was almost inhibited by siRNA. Moreover, compared with the scrambled control group, cell migration was inhibited in the Twist1 knockdown group. As GCB induced a decrease in Twist1 levels through proteasome-mediated degradation, several intracellular signaling pathways that regulate Twist1 were investigated. When the cells were treated with GCB, the phosphorylation of the ERK activation loop was inhibited (Figure 4E). The cells were treated with U0126 to check whether the decrease in Twist1 levels was affected by the reduced phosphorylation of ERK. ERK phosphorylation on Thr202/Tyr204 and Twist1 expression were downregulated by U0126 (Figure 4F). The Akt/GSK3β signaling pathway, which is also involved in Twist1 stability, was not notably altered by GCB treatment (Figure S2). Taken together, these data indicate that GCB suppresses Twist1 in HCC through the inhibition of ERK pathways.

## 3. Discussion

Natural products, generally obtained from fruits, vegetables, and herbs, have been used as medicines to treat various diseases for a long time [26]. Many natural product-derived phytochemicals have exhibited anti-cancer effects by inhibiting the initiation and development of cancer through the modulation of mechanisms such as cell cycle, apoptosis, cell proliferation, angiogenesis, and metastasis [27].

As natural products are cost-effective, easily accessible, and readily applicable to cancer therapy/chemotherapy, their use has been extensively studied for decades [26]. Various studies have reported the efficacy of GCB in inhibiting cancer growth in many carcinoma cell lines, but not in HCC cell lines [14,15,28,29,30]. GCB is considered to be a nonspecific inhibitor of transcription or translation because reduced AP-1 and nuclear factor kappa B (NF-κB) activities have been observed in NCI-60 cells treated with GCB [13]. In the present study, the cells treated with GCB showed suppressed phosphorylation of ERK. As the activities of the transcription factors, AP-1 and NF-κB, are regulated by MAPK pathways [31], the inhibition of AP-1 and NF-κB may result from the down-regulation of MAPK pathways. Another study revealed that GCB suppresses the growth of colorectal cancer cells through the inhibition of β-catenin and hypoxia-inducible factor 1-α, which is mediated by the down-regulation of the PAK1 pathway [14]. GCB in combination with gemcitabine has also been reported to reduce the growth of pancreatic cancer cells through the down-regulation of PAK1 and PAK4 [15].

The EMT-associated transcription factors Zeb1, Snail1, and Twist1 play roles in the regulation of EMT during the metastasis of cancer cells through different signaling cascades, including the Akt and ERK1/2 pathways [32,33]. We found that GCB treatment decreased the ERK1/2 phosphorylation and total Twist1 protein levels. In addition, GCB suppressed Twist1 protein levels without altering Twist1 mRNA levels, indicating that GCB alters Twist1 protein levels through the regulation of its protein stability except for its transcriptional expression. A previous study revealed that the phosphorylation of Twist1 at Ser68 induces its stabilization [12]. Ser68 of Twist1 was phosphorylated by MAPKs, including ERK, JNK, and p38. The cells treated with GCB showed the inhibition of ERK phosphorylation and Twist1 degradation, indicating that GCB inhibits the MAPK/Twist1 pathway. Thus, HCC cells with decreased Twist1 levels showed suppressed cell motility and invasion.

A significant increase in the mRNA levels of MMP-9 has been reported in cells overexpressing Twist1 [34]. In addition, a positive correlation has been reported between Twist1 and MMP-2 and -9 [35]. The overexpression of Twist1 and MMP-9 affects the overall survival and metastasis of human gastric carcinoma patients [36]. In the present study, the cells treated with GCB showed reduced MMP activities and suppressed expression levels of Twist1.

We investigated the anti-cancer effects and action mechanisms of GCB in HCC cell lines. The inhibition of the MAPK/Twist1 pathway by GCB resulted in dramatic suppression of EMT-associated cellular functions, such as cell migration, invasion, and MMP activities. Thus, GCB has a high potential as an anti-cancer agent originating from a natural product yet further investigation and development are needed.

## 4. Materials and Methods

### 4.1. Cell Culture and Reagents

Huh7 and Huh7-cHA-Snail cells were cultured in RPMI 1640 medium (WELGENE Inc., Gyeongsangbuk-do, Korea) containing 10% FBS (WELGENE Inc.) and 1% penicillin–streptomycin (Thermo Fisher Scientific, Inc., Waltham, MA, USA). Dulbecco’s modified Eagle’s medium (WELGENE Inc., Gyeongsangbuk-do, Korea) containing 10% FBS and 1% penicillin–streptomycin was used for culturing SK-Hep1 and Hep3B cells. All the cells were subcultured every 48 h at a 1:5 ratio and incubated at 37 °C under 5% CO_2_. U0126 was purchased from Sigma-Aldrich (Sigma-Aldrich, St. Louis, MO, USA).

### 4.2. Wound Healing Screening

Huh7 cells were seeded in 96-well plates at 90% confluence and then incubated overnight. A wound was created using a white tip (Axygen Scientific, Inc., Waltham, MA, USA). The cultured medium was sucked and replaced with diluted chemicals obtained from the library. All the chemicals were dissolved in DMSO (Sigma-Aldrich, St. Louis, MO, USA) and diluted to 1 μM each with 1% FBS medium. The scratched cells were treated with the chemicals for 24 h. The cells that migrated into the wound surface were observed using a JuLI Stage Real-Time Cell History Recorder (NanoEnTek, Inc., Seoul, Korea). The change in wound closure is represented as the percentage of wound recovery. All the experiments were performed in triplicate.

### 4.3. Antibodies

HA antibody (TA 150034) was purchased from OriGene Technologies (Origene Technologies Inc., Rockville, MD, USA). p-Akt1/2/3 (sc-514032), Akt1/2/3 (sc-81434), α-tubulin (sc-5286), and Twist1 (sc-81417) antibodies were purchased from Santa Cruz Biotechnology (Santa Cruz Biotechnology, Inc., Dallas, TX, USA). p-ERK (9108), ERK (9502), GSK3β (9315), and p-GSK3β (9336) were purchased from Cell Signaling Technology (Cell Signaling Technology, Inc., Danvers, MA, USA). E-cadherin (610181) antibody was purchased from BD Biosciences (BD Biosciences, San Jose, CA, USA). FLAG antibody was purchased from Sigma-Aldrich. Horseradish peroxidase (HRP)-tagged antibodies, polyclonal anti-rabbit IgG-HRP, and polyclonal anti-mouse IgG Fc-HRP were purchased from AbFrontier (Young In Frontier Co., Ltd., Seoul, Korea), while polyclonal anti-mouse IgM-HRP was purchased from Enzo Life Sciences (Enzo Life Sciences, Inc., New York, NY, USA).

### 4.4. GCB Isolation and Preparation

The dried rootbarks of *Ailanthus altissima* (Simaroubaceae) (10 kg) were soaked in methanol at room temperature (RT) for 7 days. Concentration of the solvent yielded 890 g of a dark syrupy residue, which was suspended in 20 L of water and then partitioned with an equal volume of dichloromethane to yield 190 g of a dichloromethane fraction. The dichloromethane fraction was subjected to repeated column chromatography on silica to yield 50 mg of a white amorphous powder, which was identified as GCB by the direct comparison of spectral data with existing data [37]. GCB was diluted in DMSO and directly added to the culture media. To avoid side effects, the ratio of DMSO in media never exceeded 0.1%.

### 4.5. Cell Viability Assay

Huh7 cells (4 × 10^4^ cells/well) were seeded in 96-well plates and cultured overnight. GCB was added to the medium containing 1% or 10% FBS. Cell viability was measured every 24 h using EZ-CYTOX (DOGEN, Seoul, Korea) after the cells were exposed to GCB. All the experiments were performed according to the manufacturer’s instructions.

### 4.6. Wound Healing Assay

Huh7 cells were seeded in 6-well plates (0.5–1 × 10^6^ cells/well) and cultured until they reached 90% confluence. A wound was created using a scratcher tip (0.5 mm; SPL Life Sciences, Gyeonggi-do, Korea). The detached cells were removed with phosphate-buffered saline (PBS) supplemented with media containing 1% FBS and treated with the indicated concentration of GCB for 24 h. The area of the migrated cells was observed using the wound healing screening method reported above.

### 4.7. Transwell Migration and Invasion Assay

For the Matrigel invasion assay, 24-transwell plates (pore size, 8 µM, polycarbonate membrane; SPL Life Sciences, Gyeonggi-do, Korea) were coated in the upper chamber with 30 µL of diluted Matrigel (0.5 mg/mL; BD Biosciences, San Jose, CA, USA) for 3 h. Huh7 cells (5 × 10^4^ cells/well) in 250 µL of media containing 1% FBS were added to the upper chamber with or without GCB. The lower chamber was filled with 500 µL of media containing 10% FBS. The plates were incubated at 37 °C under 5% CO_2_ for 18 h. The membranes to which the cells migrated were fixed in 4% paraformaldehyde in PBS, and the cells adhering to the upper surface of the membrane were removed using a cotton swab. The invasive cells were stained with 0.5% crystal violet. After drying, five random fields of the membrane were observed and counted by averaging the total number of cells at 100× magnification. The transwell migration assay had the same procedure as the invasion assay, except that the upper chamber was not coated with Matrigel and the incubation duration was 6 h at 37 °C under 5% CO_2_.

### 4.8. Soft Agar Colony Formation Assay

The base agar containing 0.5% low-melting-temperature agarose (Sigma-Aldrich, St. Louis, MO, USA) in 2× cell culture medium was seeded and coated on 12-well plates and incubated for 30 min at 4 °C. Huh7 cells (4 × 10^3^ cells/well) were suspended in cell culture medium containing 0.3% low-melting-temperature agarose and plated on a semisolid 0.5% agarose medium. The cell culture medium containing GCB was freshly replaced every 3 days. After incubation for 14 days at 37 °C under 5% CO_2_, the colonies were stained with 0.05% crystal violet solution for 1 h at 25 °C and washed several times with PBS. The stained samples were photographed using a digital camera (Olympus SP-350, Cam2Com).

### 4.9. Gelatin Zymography

Huh7 cells (3.5 × 10^6^ cells/well) were seeded in 6-well culture plates and incubated in serum-free medium in the presence of the indicated concentrations of GCB for 24 h. The collected medium was mixed with 5× sample buffer (2.5 mM Tris–Cl (pH 6.8), 5% sodium dodecyl sulfate (SDS), 50% glycerol, and 0.05% bromophenol blue) and separated by 10% SDS–polyacrylamide gel electrophoresis (PAGE) with 0.1% gelatin (G1393-20ML, Sigma-Aldrich, St. Louis, MO, USA). The gels were washed twice with wash buffer (50 mM Tris–HCl (pH 7.5), 0.2 M NaCl, 5 mM CaCl2, 0.1 µM ZnCl, and 2.5% Triton X-100) for 30 min at RT and then incubated in substrate buffer (50 mM Tris–HCl (pH 7.5), 0.2 M NaCl, 5 mM CaCl_2_, 0.1 µM ZnCl_2_, 0.016% NaN_3_, and 1% Triton X-100) at 37 °C for 24 h. The gels were fixed with a fixing solution (40% methanol and 10% acetic acid) for 30 min. The gels were then stained with a staining solution (0.25% Coomassie brilliant blue R-250, 45% methanol, and 10% acetic acid) for 1 h, followed by destaining with a destaining solution (5% methanol and 8% acetic acid).

### 4.10. MMP Activity Assay

Huh7 cells (3.5 × 10^6^ cells/well) were seeded in 6-well culture plates and incubated in serum-free medium in the presence of the indicated concentrations of GCB for 24 h. The collected medium was incubated with the assay system following manufacturer’s instructions (E12055, EnzChek™ Gelatinase/Collagenase Assay Kit; Thermo Fisher Scientific, Inc., Waltham, MA, USA).

### 4.11. Three-Dimensional Spheroid Invasion Assay

Huh7 cells were seeded in round bottom low-attachment 96-well plates (2 × 10^3^ cells/well) (SPL Life Sciences, Gyeonggi-do, Korea) and incubated for 3 days. After removing the media, 3 mg/mL of Matrigel (BD Biosciences, San Jose, CA, USA) was added. The plates were centrifuged at 300× *g* for 3 min at 4 °C and then incubated at 37 °C overnight. The spheroids were treated with GCB in 10% FBS medium. All images were captured using a JuLI Stage Real-Time Cell History Recorder.

### 4.12. Transfection

For transfection, cells were used at 70% confluence on the day of transfection in 6-well plates. The plasmid was combined with linear polyethylenimine (linear PEI; PolySciences, Warrington, PA, USA) at a 1:3 ratio in serum-free cell culture medium and incubated at RT for 30 min. The mixture was then added dropwise to the appropriate well, following which the cells were incubated for 48 h at 37 °C in a cell culture incubator.

### 4.13. Twist1 siRNA Transfection

The target siRNA was purchased from Bioneer (Bioneer, Daejeon, Korea). The Twist1 siRNA-specific target sequence was as follows: forward, 5′-GGA CCC AUG GUA AAA UGC A; reverse, 5′-UGC AUU UUA CCA UGG GUC C. The scrambled sequence was as follows: forward, 5′-CCU ACG CCA AUU UCG U-3′; reverse, 5′-ACG AAA UUG GUG GCG UAG G-3′ (negative control). Hep3B cells were seeded and grown to 70% confluence without antibiotics. The prepared cells were transfected with 100 nM siTwist1 using Lipofectamine 2000 (Thermo Fisher Scientific, Inc., Waltham, MA, USA). The cells were used for the experiment 48 h after transfection. The expression levels of Twist1 protein in the knockdown and scrambled control groups were confirmed by Western blotting assay.

### 4.14. Immunoblotting Analysis

Huh7 cells were seeded in 10% FBS-containing media. When the cells reached 80% confluence, they were treated with the indicated concentrations of GCB for 24 h. Total proteins were extracted in lysis buffer containing 20 mM Tris–HCl (pH 7.5), 150 mM NaCl, 1 mM EDTA, 0.5% Triton X-100, 0.5% IGEPAL, 10% glycerol, 1 mM dithiothreitol, and 1 mM PMSF. The concentrations of soluble cell lysates were measured using the Bradford Protein Assay Kit (Bio-Rad, Hercules, CA, USA). The samples were boiled at 100 °C for 5 min. Next, 20–40 μg of cell lysates were separated on 10% polyacrylamide gel and transferred onto a nitrocellulose membrane, as described. The membrane was blocked with 5% nonfat dry milk for 1 h at RT and incubated with the appropriate antibodies with 5% BSA or 5% nonfat dry milk, followed by incubation with an HRP-conjugated secondary antibody. Immunoreactive bands were visualized using an ECL system (Pierce, Outagamie, WI, USA) and a cooled charge-coupled device camera system (AE-9150, ATTO Technology, Tokyo, Japan).

### 4.15. qRT-PCR Analysis

Total RNA was extracted with Labosol solution (COSMO Genetech, Seoul, Korea). RNA (1 μg) was reverse transcribed to complementary DNA (cDNA) using the TOPscript cDNA Synthesis Kit (Enzynomics, Tokyo, Korea). qRT-PCR was performed using a CFX Connect real-time thermal cycler (Bio-Rad, Hercules, CA, USA) with iQ SYBR Green Supermix (Bio-Rad, Hercules, CA, USA). cDNA was initially denatured at 94 °C for 5 min and then amplified by repeating 39 cycles at 94 °C for 5 s and 60 °C for 30 s. Fluorescence values of the PCR amplicon were normalized using the *GAPDH* gene and are expressed as the ratio of gene expression levels by referring to the control group as 100%, according to the 2^−ΔΔCq^ method. The primer list is provided in Table S1.

### 4.16. Statistical Analyses

All the data represent three independent experiments and are expressed as the mean ± standard error of the mean (SEM). Statistical analyses were performed by one-way ANOVA and Dunnett’s multiple comparison using Prism 3.0 (GraphPad Software, San Diego, CA, USA). *p* < 0.05, *p* < 0.01, and *p* < 0.001 were considered statistically significant.

## Data Availability

The data that support the findings of this study are available from the corresponding author upon reasonable request.

## Supplementary Materials

Supplementary Materials can be found at https://www.mdpi.com/1422-0067/22/4/1700/s1.

## Abbreviations

GCB	Glaucarubinone
HCC	Hepatocellular carcinoma
ERK	Extracellular signal-regulated kinase
JNK	c-Jun N-terminal kinase
EMT	Epithelial-to-mesenchymal transition
MMP	Matrix metalloproteinase
MAPK	Mitogen-activated protein kinase
PAK	p21-activated kinase
