# Supplementary material for: Anti-Cancer Effects of Glaucarubinone in the Hepatocellular Carcinoma Cell Line Huh7 via Regulation of the Epithelial-To-Mesenchymal Transition-Associated Transcription Factor Twist1
Source: Int J Mol Sci. 2021 Feb 8;22(4):1700. doi: 10.3390/ijms22041700 (PMC7915236; doi:10.3390/ijms22041700)
Supplement: Supplementary file 1 [file ijms-22-01700-s001.pdf]

Article

Supplementary Information for

Anti-Cancer Effects of Glaucarubinone in the Hepatocellular Carcinoma Cell Line Huh7 Via Regulation of the Epithelial-to-Mesenchymal Transition-Associated Transcription Factor Twist1

Jihye Seo[1,†], Jain Ha[1,†], Eunjeong Kang[1], Haelim Yoon[1], Sewoong Lee[1], Shi Yong Ryu[2], Kwonseop Kim[3], and Sayeon Cho[1,*]

1 Laboratory of Molecular and Pharmacological Cell Biology, College of Pharmacy, Chung-Ang University, Seoul 06974, Korea; seojh0228@gmail.com (J.S.); joehalee@gmail.com (J.H.); ejaykang@gmail.com (E.K.); limtiny@naver.com (H.Y.); dltpdnd2000@naver.com (S.L.)
2 Korea Research Institute of Chemical Technology (KRICT), Daejeon 34114, Korea; syryu@krict.re.kr
3 College of Pharmacy and Research Institute for Drug Development, Chonnam National University, Gwangju 61186, Korea; koskim@jnu.ac.kr
* Correspondence: sycho@cau.ac.kr
† These authors contributed equally to this work.

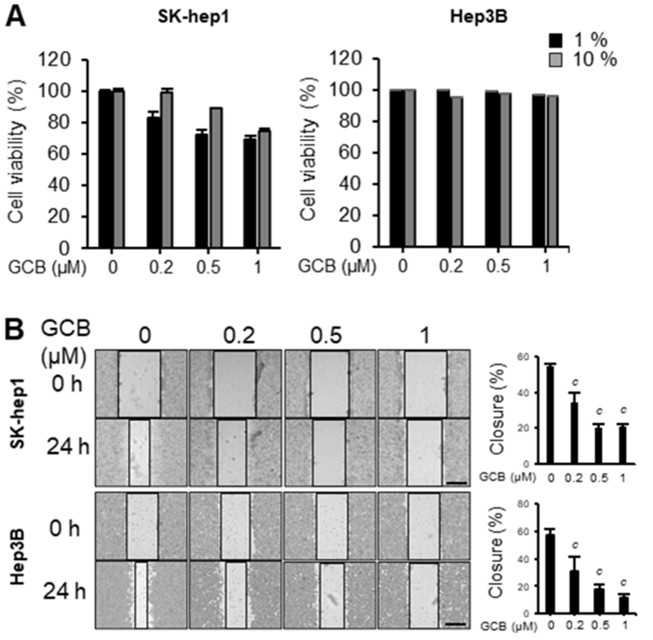

Supplementary Figure S1. Effects of GCB on other liver cancer cell lines. (A) SK-Hep1 and Hep3B cells were treated with GCB at the indicated concentration in cell culture medium containing 1% or 10% FBS for 24 h. The cell viability was measured using the EZ-CYTOX solution. The relative cell viability is shown using bar graphs in comparison with the untreated control (100%). Data are expressed as the mean ± SEM. (B) SK-Hep1 and Hep3B cells were incubated with GCB (0, 0.2, 0.5, and 1 μM). Microscopic images were taken at 24 h (scale bars, 0.5 mm). The wound closure values were quantified by measuring the percentage of wound closure in comparison with the 0 h point for each sample; the relative wound

closure is shown as a bar graph. Data were analyzed by one-way ANOVA; [c]$p < 0.001$ relative to the untreated control.

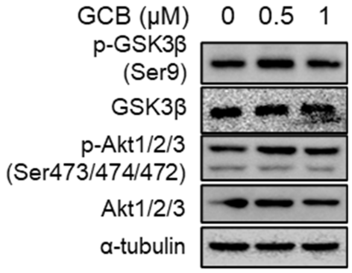

Supplementary Figure S2. Analysis of various intracellular signaling pathways. Huh7 cells treated with the indicated concentration of GCB were incubated for 24 h and then harvested. The protein samples were separated by SDS–PAGE, and the expression of other signaling pathways was evaluated using the indicated antibodies. This included p-Akt1/2/3 (Ser473/474/472), Akt1/2/3, p-GSK3β (Ser9), GSK3β, and α-tubulin. p-Akt1/2/3 is used for detection of Ser473 phosphorylated Akt1 and correspondingly Ser474 phosphorylated Akt2 and correspondingly Ser472 phosphorylated Akt3.

Supplementary Table S1. List of primers used for qRT-PCR.

**Primer Name**	**5´ to 3´**
CDH1 (F)	TACACTGCCCAGGAGCCAGA
CDH1 (R)	TGGCACCAGTGTCCGGATTA
TWIST1(F)	GGCTCAGCTACGCCTTCTC
TWIST1 (R)	CTCCTTCTCTGGAAACAATGACAT
MMP2 (F)	AGCGAGTGGATGCCGCCTTTAA
MMP2 (R)	CATTCCAGGCATCTGCGATGAG
MMP9 (F)	GCCACTACTGTGCCTTTGAGTC
MMP9 (R)	CCCTCAGAGAATCGCCAGTACT
GAPDH (F)	GCTCTCTGCTCCTCCTGTTC
GAPDH (R)	ACGACCAAATCCGTTGACTC
